# Believing Is Seeing: A Proof-of-Concept Semiexperimental Study on Using Mobile Virtual Reality to Boost the Effects of Interpretation Bias Modification for Anxiety

**DOI:** 10.2196/11517

**Published:** 2019-02-21

**Authors:** Boris Otkhmezuri, Marilisa Boffo, Panote Siriaraya, Maria Matsangidou, Reinout W Wiers, Bundy Mackintosh, Chee Siang Ang, Elske Salemink

**Affiliations:** 1 Faculty of Sciences School of Engineering and Digital Arts University of Kent Canterbury United Kingdom; 2 Faculty of Social and Behavioural Sciences Department of Psychology University of Amsterdam Amsterdam Netherlands; 3 Computer Science and Engineering Faculty of Computer Science and Engineering Kyoto Sangyo University Kyoto Japan; 4 Faculty of Science and Health Department of Psychology University of Essex Colchester United Kingdom; 5 Faculty of Social and Behavioural Sciences Department of Clinical Psychology University of Utrecht Utrecht Netherlands

**Keywords:** anxiety, emotional reactivity, interpretation bias, cognitive bias modification, virtual reality, head mounted display, immersion, presence

## Abstract

**Background:**

Cognitive Bias Modification of Interpretations (CBM-I) is a computerized intervention designed to change negatively biased interpretations of ambiguous information, which underlie and reinforce anxiety. The repetitive and monotonous features of CBM-I can negatively impact training adherence and learning processes.

**Objective:**

This proof-of-concept study aimed to examine whether performing a CBM-I training using mobile virtual reality technology (virtual reality Cognitive Bias Modification of Interpretations [VR-CBM-I]) improves training experience and effectiveness.

**Methods:**

A total of 42 students high in trait anxiety completed 1 session of either VR-CBM-I or standard CBM-I training for performance anxiety. Participants’ feelings of immersion and presence, emotional reactivity to a stressor, and changes in interpretation bias and state anxiety, were assessed.

**Results:**

The VR-CBM-I resulted in greater feelings of presence (*P*<.001, *d*=1.47) and immersion (*P*<.001, *η*_p_^2^=0.74) in the training scenarios and outperformed the standard training in effects on state anxiety (*P*<.001, *η*_p_^2^=0.3) and emotional reactivity to a stressor (*P*=.03, *η*_p_^2^=0.12). Both training varieties successfully increased the endorsement of positive interpretations (*P*<.001, *d*_repeated measures_ [d_rm_]=0.79) and decreased negative ones. (*P*<.001, d_rm_=0.72). In addition, changes in the emotional outcomes were correlated with greater feelings of immersion and presence.

**Conclusions:**

This study provided first evidence that (1) the putative working principles underlying CBM-I trainings can be translated into a virtual environment and (2) virtual reality holds promise as a tool to boost the effects of CMB-I training for highly anxious individuals while increasing users’ experience with the training application.

## Introduction

### Background

Negative biases in information processing have been found to be a vulnerability factor and to play a causal role in the development and exacerbation of emotional disorders, particularly anxiety [1-3]. Empirical evidence has shown a robust relationship between anxiety and negative interpretation bias (for a review, see the study by Hirsch et al [3]). Whereas nonanxious individuals tend to favor positive or benign interpretations of ambiguous stimuli and situations, anxious individuals favor more threatening interpretations (ie, negative interpretative bias) and tend to exaggeratedly anticipate possible negative events in the future [2,4-7]. As a result, individuals vulnerable to anxiety experience more frequent and intense emotional reactions to everyday stressors and overestimate the presence of real threats in the environment.

Experimental research established that interpretation bias can be manipulated (or *retrained*) using a scenario-based procedure labeled Cognitive Bias Modification of Interpretations (CBM-I) [4]. In this training paradigm, participants repeatedly read short text scenarios describing ambiguous situations relevant to their type of anxiety, each one ending with a word fragment. The task of the participant was to read the text and resolve the word fragment in a meaningful fashion, which, when completed correctly, can result in a positive, negative, or neutral ending. The following is an example of a training scenario:

You’ve finished writing the answer to the second question in your exam.

You take a small break, looking at what’s left.

*You then realize that the questions left are more difficult than you had anticipated. Checking the watch, you decide you’ve planned your time w_ll* [well].

A subsequent question relating to the interpretation (eg, *Will you have time to complete the exam?*) is then presented, and a training-congruent answer (*Yes* or *No*) is positively reinforced (*Correct*). Then, the next trial starts with a new scenario, and so on.

In the positive interpretation condition, the ambiguous word stem can only be completed in a benign, anxiety-irrelevant way, and participants are thus trained toward positive resolutions of the described ambiguous scenario. In the control condition, typically an equal amount of positive and negative interpretations is presented. Two meta-analyses examining the effectiveness of CBM-I as a training intervention across anxiety and depression provided evidence for both near-transfer (ie, effects on interpretation bias measured with a similar task) and far-transfer (ie, effects on emotional reactivity to stressors and/or anxiety symptoms) effects, showing small to medium effect sizes [8,9], depending on the outcome. Anxious participants who were trained to consistently make benign interpretations of ambiguous information were more likely to generalize these more benign interpretations to new ambiguous stimuli or situations. As a result, participants showed lower levels of emotional vulnerability to stress, trait and state anxiety, and symptoms of anxiety [10-16], although the results are not consistent across studies [9]. Another recent meta-analysis looking at the effects of different types of Cognitive Bias Modification (CBM) interventions concluded that CBM effects were overall small or clinically nonrelevant [17]. However, greater beneficial effects on both anxiety and depression were observed for CBM-I paradigms compared with other types of CBM trainings.

Factors have been identified that impact CBM-I effectiveness. It has been shown that CBM-I training effects are stronger when participants actively process the (corrective) information [4]. In addition, imagining the described scenarios enhanced the training effects [18]. Furthermore, CBM-I effects have been found particularly pronounced when the training involves repeated practice over multiple sessions, indicating a dose-response relationship between the number of training sessions and effectiveness [9]. However, CBM-I training tasks generally include a very basic and unattractive layout (ie, a few lines of text presented on a neutral background), which makes training sessions highly unattractive. Participants who have undergone the training have reported it to be repetitive, boring, and monotonous [19,20]. The risk is that participants get easily distracted and stop being engaged with the training, resulting in less active processing of the content of the scenarios and the crucial training contingencies and, as a result, less learning [20]. Therefore, it is paramount to optimize the functional and aesthetic features of CBM-I training tasks so as to strengthen their beneficial effects and improve training adherence.

### This Study

In this study, we tested the deployment of mobile virtual reality (VR) technology to transpose a scenario-based CBM-I training in a three-dimensional (3D) virtual environment, where the events described in the scenarios may *virtually* take place and be experienced in first person in a realistic fashion. The last two decades have seen an exponential increase in the use of VR technology in mental health treatment and within clinical research, with the greatest bulk of research showing the added benefits and long-term effects of virtual exposure therapy for different anxiety disorders, phobias, and post-traumatic stress disorder [21-25]. More recently, VR has also been extended to the adjunct treatment of psychosis, delivering cognitive rehabilitation, social skills training interventions, and VR-assisted therapies [26,27].

Furthermore, the development of information technologies has allowed mobile phones to meet all the requirements necessary to support VR (eg, appropriate central processor unit [CPU] and graphics processor unity [GPU] computing power, gyroscope integration) and, at the same time, to be portable and affordable. Their wide distribution allows more people to have access to immersive VR technology. In addition, the current generation of smartphones has become more than just devices for talking: the broadly available smartphones are capable of supporting 3D graphics, images, sounds, and software.

It is important to note that VR-based interventions and, in general, electronic health and mobile health interventions generally refer to the implementation of therapeutic principles in a digital environment rather than designing an entirely novel intervention paradigm [28,29]. In doing so, a mobile VR-based CBM-I training would harness the potential of simulating complex real-life environments where individuals can fully immerse themselves and explore, while keeping the effective principles underlying the training paradigm as intact as possible. In VR, users are no longer simply external observers of images or text on a computer screen but are active participants *immersed* in a computer-generated 3D virtual world. By introducing specific perceptual cues evoking real-life contexts where (anxiety-relevant) ambiguous situations normally occur, VR strongly relies on the activation of the emotional reactions of the same ambiguous situation experienced in the real world and potentially increases the activation of relevant threat-related cognitive schemas [1]. The emotional experience, in turn, is related to *presence*, another important concept in VR, which involves the perception of the virtual environment as being real [30], creating the user’s sense of *being* in the VR environment. As such, “VR can be described as an advanced imaginal system: an experiential form of imagery that is as effective as reality in inducing emotional responses” [31,32].

The latter feature of VR is of special interest for the optimization of CBM-I training interventions, as the use of imagery instructions in CBM-I trainings has been found to boost their effects [9]. The ability of VR to “physically” immerse users within the ambiguous scenarios and to provide the proprioceptive perception of being an active agent in the virtual world has the potential to activate relevant memory schemas and evoke the typical interpretational and emotional response. Given recent insights into the importance of (a strong) discrepancy between expectations and the actual situation [33], VR may activate the dysfunctional schema and thus enhance the discrepancy with the positive interpretation provided in the CBM-I training, boosting prediction-error learning. As such, VR has the potential to enhance the therapeutic mechanisms underlying the training intervention. In fact, the activation of (anxiety-relevant) ambiguity and the related individual’s habitual pattern of biased information processing are necessary ingredients to successfully retrain it toward a more benign resolution [4,20]. Furthermore, the interactive and immersive properties of virtual environments may lead to an improvement of motivation to engage with the training application and the overall training experience compared with other media (eg, desktop computers).

Despite VR technology being used profusely as part of exposure therapy for anxiety disorders, the use of this technological platform in other forms of psychological interventions such as CBM training has received far less attention. To the best of our knowledge, only 1 proof-of-concept study has explored the feasibility of VR-based CBM training for social anxiety targeting attentional bias for threatening stimuli [34]. Although the study did not include a control group and was not designed, nor powered, to test the effectiveness of the intervention, the VR-based attentional bias training was associated with higher scores in enjoyment, flow, presence, and motivation than the standard training, indicating good acceptance and feasibility of the VR training intervention.

The main goal of this study was to examine the feasibility of using mobile-based stereoscopic 3D VR technology in a CBM-I training paradigm (virtual reality Cognitive Bias Modification of Interpretations [VR-CBM-I]) for performance anxiety to improve the users’ experience with the training program (ie, feelings of immersion and presence) and to potentially enhance training effects on state anxiety, emotional reactivity, and interpretation bias, compared with the standard training paradigm (standard CBM-I). We hypothesized that, compared with participants receiving the standard CBM-I training, participants completing the VR-CBM-I training would show (1) higher self-reported rates of immersion and presence in the training scenarios, (2) a greater endorsement of positive interpretations and less negative interpretations after the training, (3) a greater reduction in state anxiety after the training, and (4) lower emotional reactivity to stressors.

## Methods

### Participants

Participants were recruited through convenience sampling from the undergraduate student population of the University of Kent. Candidate students were invited by email to participate in a study on the use of VR to reduce anxiety levels. A total of 67 interested students aged 18 years and above were screened online for moderate-to-high trait anxiety (a score greater than 40 on the A-Trait subscale of the State-Trait Anxiety Questionnaire, STAI [35,36],—a standardized clinical measure of trait and state anxiety) and, when meeting this criterion, further invited to schedule a lab session. A total of 42 students (23 females and 19 males) aged between 18 and 35 years (mean 21.60 [SD 2.96]) with a mean trait anxiety score of 51.0 (SD 8.7) took part in the study.

### Procedure

Upon arrival to the experimental laboratory, participants were briefly explained the goal and procedure of the study. Participants were informed that the study was focused on how CBM-I training can help support people with anxiety and that we were interested in exploring how different technologies, including VR, can facilitate the training of interpretation bias. The participants did not know the specific hypotheses of the study, nor that they would have received a stressor task. Participants were explained that they would be assigned to 2 groups of equal size, how a general scenario-based CBM-I training task worked, and that afterward they would complete general measures of stress, immersion, and system usability. After giving their informed consent, they were then assigned to either the standard CBM-I (n=21) or VR-CBM-I (n=21) training condition in a counterbalanced fashion, stratified by gender.

The experiment started with a baseline assessment of participants’ state anxiety (STAI A-State subscale) and interpretation bias (Recognition Task), followed by the training session completed on either the computer (standard CBM-I) or a head-mounted display system (VR-CBM-I) according to the allocated condition. At termination of the training and after an optional small pause, participants completed the post-training assessment of state anxiety (STAI A-State subscale), interpretation bias (Recognition Task), and perceived immersion (Immersion Experience Questionnaire [IEQ]) [37] and presence (Slater-Usoh-Steed [SUS] questionnaire) [38] during the training. The post-training assessment phase ended with a stress induction manipulation where participants rated their mood before and after performing a stressful cognitive test, the Anagram Stress Task, which has been designed to appear as an easy task to resolve but, in fact, being very difficult and almost impossible to complete, to assess their emotional response to actual failure. Finally, participants were fully debriefed about the study and the stressor procedure and compensated with a £10 voucher. The study was approved by the Research Ethics and Advisory Group of the Department of Engineering and Digital Arts of the University of Kent (reference number: 0631516).

### Training Intervention

#### Standard Cognitive Bias Modification of Interpretations

**Figure 1:**
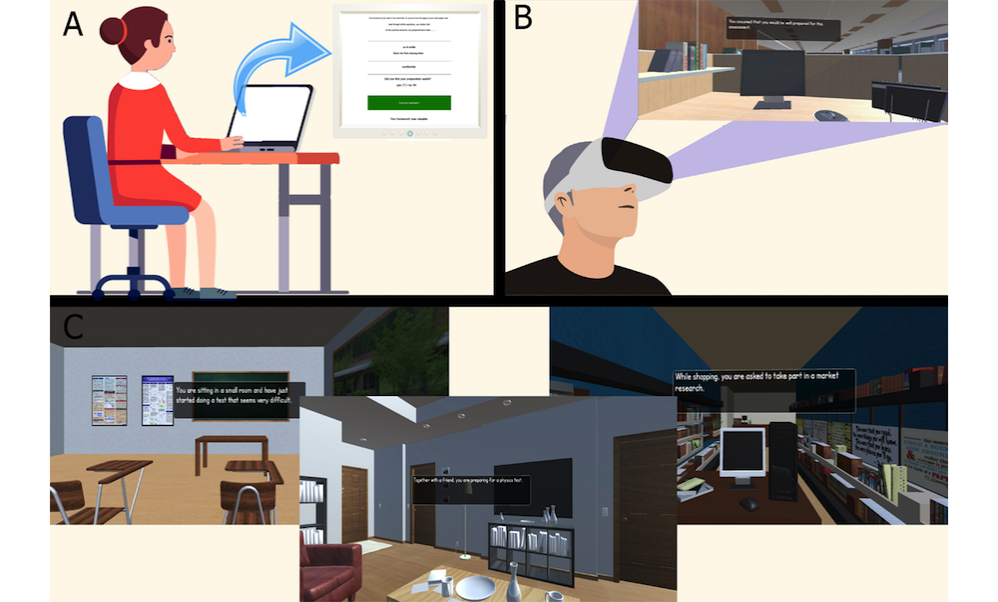
Representation of the standard Cognitive Bias Modification of Interpretation (CBM-I) and virtual reality Cognitive Bias Modification of Interpretation (VR-CBM-I) trainings: (A) Standard CBM-I training; (B) VR-CBM-I training (participant’s point of view on the computer room virtual environment on the top right corner); (C) Examples of virtual environments: classroom on the left side, living room in the middle, and book shop on the right side.

The standard CBM-I training ran on a desktop computer on E-Prime [39], with scenarios presented as plain text on a white background (see Panel A in Figure 1). Scenarios were presented in 4 blocks of 10 scenarios each, with an optional break at the end of each block. Each scenario consisted of 3 lines that were ambiguous in terms of valence. The final sentence contained a missing word. After disappearance of the scenario, the omitted word was presented as a word fragment and disambiguated the scenario in a benign, anxiety-irrelevant way. Participants were instructed to complete the word fragment as quickly and accurately as possible by pressing the spacebar and typing the first missing letter. When not knowing the answer or after 10 seconds of inactivity, the correct answer was shown on the screen. A comprehension question then appeared and participants had to reply yes or no by pressing the  *Y* or *N* button on the keyboard. Response accuracy and interpretation-relevant feedback were presented to reinforce the positive interpretation.

The scenarios were 40 event descriptions involving experiencing problems or potential failures in examination/test situations, which have been previously used in the performance anxiety domain [40]. An example of a (positive) scenario would be the following:

Together with a friend, you are preparing for a physics test.

It’s the fourth time you are discussing a topic and your friend knows more than you.

You think this is a ......

co-n-idence

coincidence

*Does your friend understand physics better than you?* (Correct response: *No*)

It was just a chance that the friend knew more than you.

#### Virtual Reality Cognitive Bias Modification of Interpretations

The VR-CBM-I training was designed to be displayed on a commercially available VR head-mounted display system (Samsung Gear VR and Samsung Galaxy S6 smartphone). Overall, 7 narrative virtual environments were created in Autodesk Maya version 2017 and Autodesk 3ds Max version 2017 software programs to represent the same 40 training scenarios used in the standard CBM-I, with each environment combining 2 to 7 scenarios (eg, exam hall, classroom, and computer room; see Panel C in Figure 1 and Multimedia Appendix 1). The stereoscopic 3D virtual environments were then textured and rendered in Unity version 5.6.0f.3, where the text in the training scenarios was added to the user interface. Participants could freely interact and explore the environment through head movements. The stories were presented as a pop-up text box appearing in the VR environment as soon as the participant started exploring it in the same presentation format as in the standard CBM-I training task (see Panel B in Figure 1). A voice recognition function that uses the inbuilt Android Speech Recognition function of the Samsung smartphone was developed and added to the VR-CBM-I system to allow participants to complete the word fragment by saying out loud the completed word and to answer the subsequent comprehension question by saying yes or no. To make the voice recording process easier and more understandable for the participants, a sound was used to indicate the start and end of the voice recognition process. Participants were able to repeat their answer again in case an incorrect word was given or recognized by the voice recognition system and were allowed to skip the step if they did not know the answer. To skip the step, participants needed to press the home button on the Samsung Gear VR glasses and the comprehension question then appeared. This design choice was made because of the different type of response input compared with the standard training, where the correct answer is normally shown automatically on the computer screen after 10 seconds when participants do not know the answer. Similar to the standard training, correct and incorrect answers to both the word fragments and the comprehension questions were visually highlighted in green and red, followed by the interpretation-congruent feedback in a pop-up text box.

### Outcome Measures

#### Immersion and Presence Experience

Participants’ subjective experience of being immersed in the training scenarios was assessed with the IEQ [37], which consists of 31 items scored on a 5-point Likert scale covering 5 aspects underlying the immersive experience with a digital environment: emotional (6 items; eg, “To what extent did you feel that the scenario was something you were experiencing, rather than something you were just doing?”) and cognitive (9 items; eg, “To what extent did you feel you were focused on the scenario?”) involvement, which refer to the feelings and the amount of focus experienced while interacting with the digital environment; real-world dissociation (7 items; eg, “To what extent did you feel as though you were separated from your real-world environment?”), which refers to the sense of detaching from the outside world and increasing awareness of the digital environment; challenge (4 items; eg, “To what extent did you find the training scenario easy?”), which is the experience of being challenged by the digital environment; and control (5 items; eg, “At any point did you find yourself become so involved that you were unaware you were even using controls?”), which is the extent to which the user feels in control while interacting with the training. The IEQ was originally designed for the serious games field and has shown acceptable psychometric properties [41]. To adapt it to the context of this study, all game-related instances in the items were replaced with “involvement with the training scenarios.”

The experience of presence within the training scenarios was assessed with the SUS [38], a 6-item questionnaire rated on a 7-point Likert scale evaluating (1) the sense of *being there* in the scenarios as compared with being in a place in the real world (eg, “Please rate your sense of being in the scenario, on the following scale from 1 to 7, where 7 represents your normal experience of being in a place.  *I had a sense of “being there” in the scenario.*”), (2) how much the scenarios became the dominant reality (eg, “To what extent were there times during the experience when the scenario was the reality for you? *There were times during the experience when the scenario was the reality for me...*”), and (3) the extent to which a participant remembered the scenarios as a place visited, rather than as a computer-generated text or image (eg, “When you think back about your experience, do you think of the scenario more as ‘images’ that you saw, or more as somewhere that you visited?  *The scenario seems to me to be more like…*”). Originally designed in the VR field, the questionnaire has been tested in multiple empirical studies and has shown to correlate with behavioral measures of presence [38,42]. For the purpose of this study, all VR instances in the questionnaire were carefully replaced with *scenarios*.

#### Interpretation Bias

Positive and negative interpretations were assessed with the Recognition Task before and after the training, a validated computerized task that has shown great sensitivity in capturing CBM-I training effects across both subclinical and clinical samples [4,12-14].

The task is similar in structure to the scenario-based standard CBM-I training (only with an added title), yet both the solution of the word fragment and the comprehension question do not disambiguate the scenario, which remained ambiguous. The task presented 10 new, unique ambiguous scenarios related to performance anxiety at each assessment timepoint. An example of a test scenario is the following:

Facts and Logic

You are working through a set of examples in your exam and

concentrating very hard to try and remember the facts and logic you studied earlier.

When it comes to recalling what you havelearnt

you feel you know how effectively the test measures your true ......

m--ory ability

memory ability

Was your memory for facts and logic being tested in an exam?

After presenting the 10 scenarios in a random order, the titles of the scenarios with 4 interpretations were presented again one at a time in random order. Participants were asked to rate the 4 interpretations on a 1 (“very different *”*) to 4 (“very similar *”*) scale for how similar in meaning each was to the original one [12,14]. The sentences represented (1) a possible positive interpretation, (2) a possible negative interpretation, (3) a positive foil sentence, and (4) a negative foil sentence. The 4 corresponding sentences of the *Facts-and-logic*-scenario are presented here as follows:

Facts and logic

1. You think you did not do well in the test because you cannot apply your good memory ability.

2. You think you will do well in the test because good memory is not important for it.

3. You think you will not do well in the test revealing your poor memory ability.

4. You think you will do well in the test because of your good memory ability.

#### Emotional Outcomes

State anxiety was assessed with the A-State subscale of the STAI questionnaire Form Y [35], which is a standardized measure of subclinical and clinical trait (A-Trait subscale) and state (A-State subscale) anxiety with very robust psychometric properties [36], including 20 items rated on a 4-point Likert scale. Stress reactivity to failure was measured by assessing participants’ emotional responses to a cognitive stressor, the Anagram Stress Task [43]. Participants were presented with 13 anagrams of different levels of difficulty that had to be solved within 28 seconds by typing the correct word. A new anagram was presented after responding or when the 28 seconds were expired. Participants were told that the task was a test of their language skills, which were found to be a reliable predictor of success in many domains, and that students normally perform well in such a task. Although the test appeared relatively easy, it was in fact extremely difficult, so that all participants failed most items. Before and after the task, participants rated how anxious and how sad they felt on 2 visual analogue scales (VAS) ranging from 1 (“happy” or “relaxed”) to 100 (“sad” or “anxious”).

## Results

### Sample Descriptives

Table 1 shows baseline sample descriptives. Comparison between the groups revealed no significant baseline differences in age, gender, trait and state anxiety, previous experience with VR, or accuracy in the solution of both the word fragments and the comprehension questions in the pretraining Recognition Task.

### Presence and Immersion

An independent-samples *t* test was carried out to examine whether participants completing the VR-CBM-I experienced more intense feelings of presence during training than participants completing the standard CBM-I training, as measured by the mean rating on the SUS items. Results showed that the VR-CBM-I group experienced significantly higher levels of presence (mean 4.97 [SD 0.90]) than the standard CBM-I group (mean 3.33 [SD 1.30]; *t*_40_=4.75, *P*<.001, *d*=1.47).

To test whether the VR-CBM-I condition was associated with a more immersive experience than the standard CBM-I condition, a multivariate analysis of variance was carried out using the 5 IEQ subscales. A significant main effect of Group was observed (*F*_5,36_=20.9, *P*<.001, *η*_p_^2^=0.74), indicating that the VR-CBM-I group experienced a greater degree of immersion in the training scenarios than the standard CBM-I group. Univariate analyses indicated that the VR-CBM-I and standard CBM-I groups differed significantly on the following 4 subscales, control, real-world dislocation, emotional involvement, and cognitive involvement, and not on the challenge subscale (see Table 2).

**Table 1:** Sample descriptives at baseline: group means (SD) or frequencies (%), statistics, *P* value, and measure of effect size (Cohen *d* or Cramer *V*).

Variables		VR-CBM-I^a^	Standard CBM-I^b^	Statistics		*P* value	Effect size	
				χ^2^ value (*df*)	*t* value (*df*)		Cohen *d*	Cramer V
**Gender, n (%)**								
	Males	7 (16.7)	12 (28.6)	0.24 (1)	N/A^c^	.12	N/A	0.24
	Females	14 (33.3)	9 (21.4)	0.24 (1)	N/A	.12	N/A	0.24
Age, mean (SD)		21.05 (1.91)	22.14 (3.7)	N/A	−1.20 (40)	.24	0.37	N/A
Trait anxiety, mean (SD)		50.43 (8.63)	51.57 (8.93)	N/A	−0.42 (40)	.68	0.13	N/A
State anxiety, mean (SD)		45.76 (6.30)	44.48 (4.66)	N/A	0.75 (40)	.46	0.23	N/A
**Baseline accuracy recognition task, mean (SD)**								
	Word fragments	0.76 (0.77)	1.10 (0.77)	N/A	−1.41 (40)	.17	0.39	N/A
	Comprehension questions	1.86 (1.46)	1.96 (1.28)	N/A	−0.23 (40)	.82	0.07	N/A
**Previous experience with VR^d^** **, n (%)**								
	Yes	4 (9.5)	3 (7.1)	0.17 (1)	N/A	.68	N/A	0.06
	No	17 (40.5)	18 (42.9)	0.17 (1)	N/A	.68	N/A	0.06

^a^VR-CBM-I: virtual reality Cognitive Bias Modification of Interpretations.

^b^CBM-I: standard Cognitive Bias Modification of Interpretations.

^c^N/A: not applicable.

^d^VR: virtual reality.

**Table 2:** Mean scores for the Immersive Experience Questionnaire subscales (SD in parentheses), *F* statistics, *P* value, and effect size (η^2^_p_) for the VR-CBM-I and standard CBM-I groups.

IEQ^a^ subscale	VR-CBM-I^b^, mean (SD)	Standard CBM-I^c^, mean (SD)	*F* statistics (*F*_1,40_)	*P* value	η^2^_p_
Challenge	4.18 (0.79)	3.96 (0.80)	0.77	.39	0.02
Control	4.85 (0.86)	3.32 (0.84)	33.73	<.001	0.46
Real world dislocation	5.14 (0.52)	3.03 (0.81)	100.33	<.001	0.72
Emotional involvement	4.49 (0.95)	3.10 (0.77)	26.86	<.001	0.40
Cognitive involvement	5.34 (0.57)	4.26 (0.70)	30.26	<.001	0.43

^a^IEQ: Immersion Experience Questionnaire.

^b^VR-CBM-I: virtual reality Cognitive Bias Modification of Interpretations.

^c^CBM-I: standard Cognitive Bias Modification of Interpretations.

### Interpretation Bias

To test whether the VR-CBM-I training was more effective in changing interpretations than the standard CBM-I training, the Recognition Task data were subjected to a 2×2×2×2 mixed analysis of variance (ANOVA) with Group (VR-CBM-I vs standard CBM-I) as between-subjects factor and Time (pre- vs post-training), Valence (positive vs negative), and Interpretation type (Target vs Foil) as within-subject factors. A significant main effect of Interpretation type was revealed (*F*_1,40_=71.0, *P*<.001, *η*_p_^2^=0.64), as well as 2 significant 2-way interaction effects (Time×Valence, *F*_1,40_=36.3, *P*<.001, *η*_p_^2^=0.48; and Time×Interpretation type, *F*_1,40_=7.3, *P*=.01, *η*_p_^2^=0.15). These effects were subsumed within a significant higher order 3-way interaction effect of Time×Valence×Interpretation type (*F*_1,40_=8.2, *P*=.007, *η*_p_^2^=0.17). To decompose the 3-way interaction effect, separate analyses were carried out for target and foil sentences (Interpretation type) separately. Both analyses revealed significant Time×Valence interaction effects (Targets: *F*_1,40_=36.0, *P*<.001, *η*_p_^2^=0.47; Foils: *F*_1,40_=10.0, *P*<.001, *η*_p_^2^=0.20). The effects sizes for these interaction effects were larger for the targets compared with the foils, suggesting stronger training effects on interpretations than on foil statements. Subsequently, separate pairwise *t* tests were conducted to decompose the Time× Valence effects for targets and foils separately. Consistent with the goal of the positive interpretation training conditions, there was a significant increase in positive target interpretations (*t*_41_=−5.1; *P*<.001; d_rm_=0.79; pretraining: mean 2.16 [SD 0.40]; post-training: mean 2.50 [SD 0.44]) and a significant decrease in negative target interpretations (*t*_41_=4.7; *P*<.001; d_rm_=0.72; pretraining: mean 2.44 [SD 0.44]; post-training: mean 2.07 [SD 0.50]). The effects were less pronounced for the foils, and only the increase in the endorsement of positive foil sentences was significant (*t*_41_=−5.2; *P*<.001; d_rm_=0.81; pretraining: mean 1.95 [SD 0.40]; post-training: mean 2.24 [SD 0.44]; negative foil sentences, *t*_41_=0.3; *P*=.76; d_rm_=.04; pretraining: mean 1.95 [SD 0.48]; post-training: mean 1.93 [SD 0.48]). Collectively, this suggests that the stronger training effects on targets versus foil sentences are driven by the specificity effects in the negative interpretations. The 4-way interaction effect of Group×Time×Valence×Interpretation type was not significant (*F*_1,40_=0.9, *P*=.35, *η*_p_^2^=0.02), indicating that the VR-CBM-I training did not result in stronger effects on interpretations than the standard CBM-I training.

### State Anxiety

**Figure 2:**
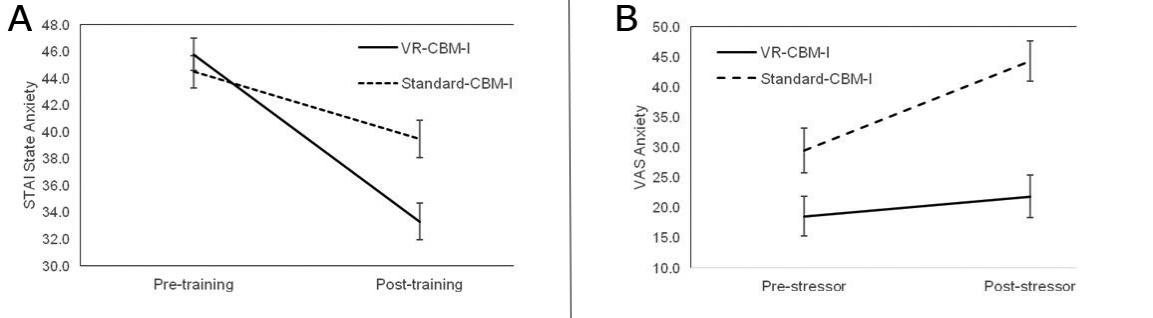
(A) Mean (and SE) state anxiety scores from pre to post-training for the virtual reality Cognitive Bias Modification of Interpretation (VR-CBM-I) and standard Cognitive Bias Modification of Interpretation (CBM-I) groups. (B) Mean (and SE) VAS anxiety scores from pre- to post-stressor for the VR-CBM-I condition and standard CBM-I condition.

To test whether the VR-CBM-I training resulted in a stronger reduction in state anxiety than the standard CBM-I training, the STAI A-State scores were subjected to a 2 (Group: VR-CBM-I vs standard CBM-I training) ×2 (Time: pre- vs post-training assessment) mixed ANOVA. There was a significant main effect of Time (*F*_1,40_=120.9, *P*<.001, *η*_p_^2^=0.75) and a significant Group×Time interaction effect (*F*_1,40_=22.0, *P*<.001, *η*_p_^2^=0.35), confirming the stronger effects of the VR-CBM-I on anxiety. That is, although state anxiety did not differ significantly between the 2 groups before training (*t*_40_=0.8, *P*=.46, *d*=0.23), participants who completed the VR-CBM-I training reported significantly less anxiety symptoms after training than participants in the standard CBM-I group (*t*_40_=−3.1, *P*=.003, *d*=0.97; see Panel A in Figure 2).

### Stress Reactivity

To test whether the VR-CBM-I training resulted in a reduced emotional response to the stressor, the VAS Anxiety was subjected to a 2 (Group: VR-CBM-I vs standard CBM-I training) ×2 (Time: pre- vs post-stressor) mixed ANOVA. In addition to significant main effects of Time (*F*_1,40_=12.9, *P*=.001, *η*_p_^2^=0.24; increase in anxiety from pre- to poststressor) and Group (*F*_1,40_=15.4, *P*<.001, *η*_p_^2^=0.28; lower anxiety in the VR-CBM-I group), the predicted Group×Time interaction effect was significant (*F*_1,40_=5.2, *P*=.03, *η*_p_^2^=0.12). Consistent with our predictions, the stress task resulted in a significant increase in anxiety in the standard CBM-I group (*t*_20_=−3.3, *P*=.003, *d*=0.72), but this was not the case for the participants who followed the VR-CBM-I training (*t*_20_=−1.4, *P*=.18, *d*=0.31; Panel B in Figure 2).

Exploratively, we examined whether the effects of training on emotional reactivity generalized to depressive feelings by subjecting the VAS Sadness to the same 2×2 mixed ANOVA. Again, significant main effects of Time (*F*_1,40_=41.8, *P*<.001, *η*_p_^2^=0.51; significant increase in sadness from pre- to post-stressor) and Group (*F*_1,40_=12.2, *P*=.001, *η*_p_^2^=0.23; lower sadness scores in the VR-CBM-I group) were observed. However, the Group×Time interaction effect was not significant (*F*_1,40_=2.7, *P*=.09, *η*_p_^2^=0.07).

### Posthoc Analyses

To examine whether the observed changes in state anxiety and emotional reactivity to the stressor were associated with perceived immersion and presence, Pearson correlations were computed between the IEQ and SUS scores and changes in state anxiety over the course of the training and changes in anxiety reactivity due to the stressor (see Table 3). Change indices were calculated by subtracting pretraining from post-training scores (ie, negative values indicate greater decrease). Stronger reduction in state anxiety across the training was significantly correlated with higher control, real-world dislocation, emotional involvement, and cognitive involvement. Furthermore, less anxiety reactivity was significantly correlated with greater perceptions of real-world dislocation and cognitive involvement.

**Table 3:** Pearson correlation coefficients between Immersion Experience questionnaire and Slater-Usoh-Steed questionnaire scores and changes in state anxiety and in anxiety reactivity due to the stressor.

Emotional Outcomes	IEQ^a^ challenge	IEQ control	IEQ dislocation	IEQ emotional involvement	IEQ cognitive involvement	SUS^b^
State anxiety change	−0.10	−0.49^c^	−0.51^c^	−0.33^d^	−0.52^e^	−0.18
Anxiety reactivity to stressor	−0.16	−0.26	−0.36^d^	−0.17	−0.31^d^	−0.12

^a^IEQ: Immersion Experience questionnaire.

^b^SUS: Slater-Usoh-Steed questionnaire.

^c^*P*<.01.

^d^*P*<.05.

^e^*P*<.001.

We further computed the correlation between changes in interpretation bias scores (amount of positive target interpretations and­ amount of negative target interpretations) as a result of the training intervention (difference in interpretation bias score between post-training and pretraining) with changes in state anxiety and anxiety reactivity and with the IEQ and SUS scores. None of the correlations were significant (Pearson *r* range=[−0.13 to 0.20], *P*>.05).

## Discussion

### Principal Findings

In this proof-of-principle study, we examined the use of stereoscopic 3D VR technology to enrich the training experience and ultimately enhance the effects of CBM-I training for performance anxiety. The main idea behind the study was that embedding the training scenarios in a virtual environment where participants could immerse themselves and explore would improve the participants’ engagement with the training and amplify the activation of (anxiety-relevant) schemas and the related individual’s habitual pattern of biased information processing (ie, negative interpretation bias), which are necessary ingredients for this type of intervention to succeed.

When examining participants’ experience with the training, the VR-CBM-I group experienced a higher degree of immersion and presence during the training than the standard CBM-I group. In particular, the results showed that there was a significantly higher level of perceived control, real-world dissociation, and emotional and cognitive involvement for participants in the VR-CBM-I group, whereas there was no significant group difference in the level of perceived challenge. Consistent with previous studies where standard CBM-I interventions have shown to reduce state anxiety levels [11-13], all participants showed an overall decline in state anxiety after the training. As hypothesized, these reductions were significantly more pronounced in the VR-CBM-I group compared with the standard CBM-I. In addition, lower anxiety reactivity to a stressor was observed in the VR-CBM-I compared with the standard CBM-I group. Nevertheless, contrary to our expectations, there was no significant difference between the 2 training versions in the impact of the training on the target information processing mechanisms, as both versions resulted in a comparable increase in positive interpretations and a decrease in negative ones.

Posthoc analyses showed that a higher degree of cognitive involvement in the training scenarios and a greater perception of dissociation from the outside real world were related to both a greater reduction in state anxiety and lower anxiety reactivity to the stressor. Furthermore, a greater feeling of emotional involvement and being in control within the scenarios were also positively associated with reductions in state anxiety. Conversely, greater feelings of presence were not associated with any change in state anxiety or emotional reactivity.

Altogether, the results of the study seem to suggest a combination of specific and nonspecific effects of the VR-based CBM-I training on anxiety. The 2 versions of the training did not differ in the successful manipulation of the targeted interpretation bias for threatening information: all participants showed a decrease in the tendency to interpret ambiguous information negatively in favor of more benign interpretations. Furthermore, although both groups showed a decrease in state anxiety, VR-CBM-I training induced a steeper reduction in state anxiety and a blunted emotional response to the stressor. Supposedly, the combination of the CBM-I training mechanisms and other VR-specific factors may have enhanced these effects. Although to be taken cautiously, the positive correlations between changes in state anxiety and anxiety stress reactivity and the control, cognitive and emotional involvement, and real-world dissociation components of the immersive experience in the virtual environment seem to support this hypothesis. By experiencing the scenarios in a *deeper* fashion—hence, by more effectively activating the biased threat-related interpretive schemata—the training effects on basic information cognitive processing would more easily generalize to stronger emotional effects, as observed in the VR-CBM-I group.

### Limitations

Despite the very promising results, no definite conclusion on the (clinical) effectiveness of VR-CBM-I can currently be drawn. Being the very first combining VR and CBM-I, this study was primarily concerned with examining the feasibility and potential of VR-CBM-I training, by focusing, as a first step, on comparing the delivery modes of the training within a semiexperimental design. Therefore, the lack of a full control condition (ie, a placebo or neutral CBM-I training group) prevents from claiming that VR-CBM-I is *more* effective than the standard CBM-I. The next step in the evaluation of VR-CBM-I would consequentially involve a full factorial experimental design, combining the 2 delivery modalities (VR yes vs no) and the 2 intervention components (active vs neutral CBM-I), to (1) experimentally compare the effects of both interventions against a neutral condition with no active training ingredients and (2) disentangle the active effects of the VR environment from the CBM-I training specific effects.

Relatedly, according to the preliminary phase of the study, participants completed only 1 session of training in the lab. Although the VR-based CBM-I successfully impacted on emotional outcomes in the immediate term and in response to a stressor, the duration of the effects over time is yet to be tested against a full control condition, as well as the exposure to multiple sessions of training over time. These latter aspects are particularly crucial in the view of effectively deploying (mobile) VR-based CBM interventions. The findings of this study are also promising regarding the boredom participants experience with multiple sessions of standard CBM-I [18,19].

The results of our study are restricted to the type of anxiety considered (ie, performance anxiety) and the self-selected group of undergraduate university students based on convenience sampling. Although students actively responded to flyers advertising the training as a tool they could use to do something for their test stress and anxiety, they were all compensated for participation, which might have involved an exaggeration of their initial levels of trait anxiety to be included in the study. Relatedly, the preliminary nature of the study involved recruiting only a small sample, resulting in an overall lack of power for the generalization of the behavior-change effects of the VR-CBM-I. Whether the results of this study may be generalized to other forms of (more severe) anxiety and groups of patients will need to be further investigated in a larger study with a self-motivated target population (eg, patients with anxiety problems).

Finally, the study points us to a number of key design questions. First, within the scope of this experiment, it is not yet clear how or to what extent the various perceptual factors within the 3D virtual environment (eg, the 3D background view, ambient noises, animation, and blur) influenced the outcomes of the VR-CBM-I training. From a design perspective, the deployment of highly controlled and more sophisticated experimental designs would allow us to achieve a greater insight to further optimize the mobile VR training intervention, allowing us to isolate and compare the effects of different technical features on the users’ perception of the virtual environment and the working mechanisms of the intervention (eg, trials of intervention principles [44]). For example, in this study, the training scenarios were embedded in the corresponding virtual environment as pop-up text appearing on the user’s visual field, which could be perceived as being *artificial* or not realistic enough. The use of audio narration of the scenarios may be a feasible option in the future development of the VR system to enhance both the training experience and the activation of the targeted emotional response [45,46]. Furthermore, given that the user interactions within the current mobile VR system were restricted to the presentation of premade scenarios and situations, future VR-CBM-I developments could investigate the use of a more interactive mobile VR system, allowing the scenarios, situations, and environments to unfold based on the choices and actions of the users. This could potentially afford a larger degree of freedom to explore and interact with the computer-generated virtual space, which could more effectively mirror users’ (emotional) experience and interaction with their real daily environment.

### Conclusions

To conclude, this proof-of-principle study is the first investigating the feasibility and potential of using mobile VR technology to deliver CBM-I training for anxiety problems. When compared with the standard CBM-I training, a mobile VR-based CBM-I training improved the users’ experience with the training program and produced greater beneficial effects on anxiety-related emotional outcomes, while similarly changing the targeted cognitive processes. This study provided first evidence that (1) the putative working principles underlying CBM-I trainings can be translated into a virtual environment, and (2) stereoscopic 3D mobile VR technology appears to be a promising technological affordance to boost the effects of such a class of interventions, while increasing users’ experience with the training application.

## Appendix

Multimedia Appendix 1 Video showing some examples of the virtual environments included in the virtual reality Cognitive Bias Modification of Interpretations training intervention.

## Abbreviations

ANOVA: analysis of variance
CBM: Cognitive Bias Modification
CBM-I: Cognitive Bias Modification of Interpretations
drm: drepeated measures
IEQ: Immersion Experience Questionnaire
STAI: State-Trait Anxiety
SUS: Slater-Usoh-Steed
VAS: visual analogue scales
VR: virtual reality
VR-CBM-I: virtual reality Cognitive Bias Modification of Interpretations
3D: three-dimensional
